# Better safe than sorry: Evaluating the implementation process of a home-visitation intervention aimed at preventing unintentional childhood injuries in the hospital setting

**DOI:** 10.3389/frhs.2022.944367

**Published:** 2022-09-02

**Authors:** Ligat Shalev, Mary C. J. Rudolf, Sivan Spitzer

**Affiliations:** Azrieli Faculty of Medicine, Bar-Ilan University, Safed, Israel

**Keywords:** hospital-based intervention, pre-school children, home safety, injury prevention, home visit, implementation science, Consolidated Framework for Implementation Research

## Abstract

**Background:**

Child home injuries prevention interventions have rarely been implemented in hospitals. The SHABI program (“Keeping our Children Safe”; in Hebrew: “SHomrim Al BetIchut Yeladenu”) recruits at-risk families arriving with child injury to the Emergency Department. Medical/nursing students conduct two home visits four months apart, providing safety equipment and guidance. One hundred thirty-five families had a first visit and 98 completed the second. Fifty percentage of families were ultra-Orthodox Jews, 11% Arab, and 28% had ≥3 preschool children. We investigated SHABI's implementation using the Consolidated Framework for Implementation Research (CFIR).

**Methods:**

Between May 2018 and March 2021 SHABI was implemented in the Emergency Department of a hospital in Israel's northern periphery, an area with high child injury rates. The Implementation process was examined through Emergency Department medical records and tracking registries, hospital management, nurses', and home visitors' meetings notes (*n* = 9), and a research diary. Hospital's inner setting and SHABI's characteristics were evaluated through interviews with hospital management, nurses, and home visitors 8 months after baseline (*n* = 18). Home visitors' characteristics were evaluated through interviews, post-visit questionnaire on challenges encountered (*n* = 233), families' perceptions of SHABI and home visitors' skills through telephone interviews (*n* = 212); and home visitors awareness of dangers at home (*n* = 8) baseline and 8 months later. Qualitative data were analyzed through explanatory content analysis according to CFIR constructs. Quantitative data were analyzed using X2 and Wilcoxon test for dependent subgroups.

**Results:**

Despite alignment between SHABI and the hospital's mission, structural hospital-community disconnect prevented implementation as planned, requiring adaptation and collaboration with the medical school to overcome this barrier. Recruitment was included in the initial patient triage process but was only partially successful. Medical/nursing students were recruited as home visitors, and following training proved competent. Children were a distraction during the visits, but home visitors developed strategies to overcome this.

**Conclusions:**

Injury prevention programs in hospitals have significant benefits. Identifying implementation barriers and facilitators allowed implementers to make adaptations and cope with the innovative implementation setting. Models of cooperation between hospital, community and other clinical settings should be further examined.

## Introduction

Unintentional childhood injuries are a major worldwide health and healthcare concern (1–3). In the United States, almost two million children <5 years old are admitted annually to the Emergency Department following an unintentional injury (4). A sibling's previous admission due to an injury poses additional risk for a child's arrival at the Emergency Department for an injury (5). Yet, many of the injuries occurring in the home environment could have been prevented by improving home safety and increasing parental supervision (6, 7).

Over the years, a leading strategy for unintentional injury prevention employs parental guidance through home-visitation (8). Such interventions have been implemented mainly in community settings, such as primary care clinics (9, 10) or early childhood centers (11, 12). Interestingly, despite Emergency Department admittance rates and hospitals being central stakeholders for reducing child injuries, their role in injury prevention has been minimal and remains unclear.

Hospitals' perceptions on recurrent visits due to disease differ. Traditionally, interaction between the patient and hospital starts with seeking care for an illness, continues with treatment provided by the hospital, and results in recovery and discharge; once a patient is discharged, hospital responsibilities cease (13). In recent years, there have been efforts to reduce recurrent hospital visits for both adults (14) and children (15). This includes an expansion in hospital care models involving the community setting through staff home visits or follow-up phone calls after discharge (16). Although recurrent visits due to child injuries remain a pressing matter, little has changed regarding hospital outreach to prevent avoidable hospital visits due to child injury.

Evidence regarding hospital leadership in designing and implementing home-visitation interventions for reducing child injury is particularly lacking. A literature search reveals only one study reporting a hospital-led intervention where families were approached 3 days post-hospital discharge following a child's injury (17). One thousand one hundred and seventy-two families received two home visits 1 year apart and two follow-up phone calls in the interim by a home visitor whose professional qualifications were not reported. While the control group received only a general safety pamphlet, the intervention group received an information pack on injury prevention; instructions by a home visitor on how to correct unsafe practices observed in the home, e.g., child's reaching small objects or lack of a smoke detector; instructions on how to prevent similar injuries to what was reported; and coupons to purchase safety devices including installation information. Findings showed no change in child injury rates between the control and intervention groups, nor significant change in parents' awareness and knowledge about child injury. Parents succeeded in improving, on average, only two unsafe practices out of the 11 measured (17). Another study recruited families to a home-visitation program from a hospital pediatric continuity clinic and focused on parental guidance on child injury prevention (18). However, recruitment from the clinic's logs included arrivals for any reason-an illness or an injury. Two further studies reported recruiting families to a home-visitation intervention *via* hospital medical records (19, 20), however their focus was improving child development and parenting practices, and home safety and child injury reduction were only secondary outcome measures.

Interestingly, a common thread in all the studies reviewed is that while the hospitals provided contact details of families *via* electronic medical records, the extent of their responsibility and involvement remained vague. Moreover, these studies are limited in their reporting of the design and implementation process of hospital-led interventions, and none to date have evaluated the possible reasons for success or failure in achieving the desired outcome in injury reduction. The implementation process of such interventions remains a “black box”. There is a need for understanding processual levers and barriers that can assist in successful implementation in a variety of contexts and settings, and which in turn could contribute to reducing recurrent hospital visits due to child injury.

In the past decade, Implementation Science has emerged as a new field of inquiry to better understand the complexities of translating evidence-based interventions into every-day practice in real-world settings (21, 22). Complexities manifest also when implementing an intervention in different contexts and settings (22). Implementation Science helps in scaling-up successful interventions, and in choosing the best approach by understanding the factors that influence the implementation process (23, 24). Further, when interventions are implemented two potentially conflicting forces may act simultaneously-fidelity vs. adaptability. Fidelity is the degree to which an intervention is implemented according to the original design, and adaptability is the extent to which an intervention may need adjustment according to setting, context, or facing barriers (25).

To date, few published studies have used the lens of implementation science to examine implementation efforts focused on reducing child injury through home visits (26, 27). Nicks et al. (26) examined the implementation process of altering a computer-based intervention into home-visitation design. The software identified home injury dangers according to the data inserted by families. In their study they evaluated the facilitators and barriers encountered, but their findings were limited to the process of altering a computer-based program into a home visit design, and not on the levers and barriers in conducting the home visits. Smithson et al. (27) conducted a systematic review for identifying facilitators and barriers for injury prevention from the perspective of community leaders, counselors, implementers, and families. While their study contributed to the identification of levers and barriers affecting the implementation process, this study did not specifically examine home-based interventions, and therefore its insights are limited.

The present study aimed to understand the barriers and facilitators to implementing a novel hospital-led intervention for reducing child injury through home visits.

### The SHABI program

SHABI (“Keeping our Children Safe”; in Hebrew: “SHomrim Al BetIchut Yeladenu”) is a program delivered in a hospital setting. Families are recruited by the pediatric Emergency Department nursing team when attending with an injured pre-school child. They are then assigned to a home visitor-a nursing or medical student, for two home visits-the first immediately following the hospital visit and the second 4 months later. The visits include a tour through the home accompanied by the parents, joint discussion on child safety in each area of the home with a checklist developed from “Beterem-Safe Kids Israel” (28), and installation of provided safety equipment. Two months later, the home visitor calls the family and offers further injury prevention guidance. The second home visit includes an additional home tour and guidance.

The students are trained in five sessions led by various experts conducted over 11 months, involving an injury prevention expert, a local ultra-Orthodox Jewish Rabbi, and the head of social services in a local Arab village. The training includes topics such as child injury epidemiology and prevention, relationship-building, cultural competence skills, and guidance on adapting the visit to the family's culture.

### The conceptual framework used in this study

To evaluate the factors affecting the implementation process, such as organizational factors and the effect implementers had, we used the Consolidated Framework for Implementation Research (CFIR) (29). CFIR was chosen as it is one of the foremost conceptual frameworks in the field of Implementation Science due to its integration of relevant theories into one unified model (29). CFIR was contextualized to assist in exploring the factors that influenced SHABI's implementation in the hospital setting, namely: (1) *Implementation process*- Assessing the intervention's planning and execution, followed by feedback and evaluation process (e.g., pre-implementation meetings); (2) *Inner setting*- Identifying the organizational factors that affect the intervention implemented (e.g., the organizational vision); (3) *Intervention characteristics*-Understanding the implementers' perceptions about the intervention (e.g., advantages or difficulties in execution); (4) *Individual characteristics*-Implementers' knowledge, opinions and skills; (5) *outer setting*-Examining the contextual factors such as regulations or policies (e.g., federal or national policies) (29). This last domain was not investigated as it was outside the study's scope.

## Methods

### Study design and setting

The study was conducted from May 2018 to March 2021 in the Pediatric Emergency Department of a hospital with 330 beds, located in Israel's northern social-geographic periphery. The hospital's surrounding towns and villages rank low in socio-economic status (SES), with 170,000 residents from diverse Jewish and Arab communities, of whom 10% are 0–4 years old (30). The area is characterized by higher rates of admissions, mortality, and attendance for unintentional childhood injuries compared with the national average (31). Intervention design and pre-implementation meetings were conducted from May 2018 to April 2019, and SHABI was delivered from May 2019 to June 2020. A significant improvement in home-safety items was observed 4 months after the first visit [14 (IQR 12–16)] vs. [17 (IQR 15–19); *p* < 0.001], accompanied by an overall increase in home safety (Mean ± SD 71.9 ± 9.5% vs. 87.1 ± 8.6%; *p* < 0.001) (32). We have reported SHABI's impact on home safety previously (32).

### Participants and procedures

The study involved the following participants:

- The hospital team-The hospital director, head of nursing, head nurse of the Emergency Department, nine Emergency Department nurses, and SHABI coordinator appointed from the hospital supervision staff.
- Home visitor team-Eleven trained nursing and medical students who conducted the home visits were paid a modest stipend per visit.
- Families who participated in SHABI-Families with adequate spoken Hebrew living in the hospital's catchment area who arrived at the Emergency Department with a <5 year old child following a home injury. One hundred thirty-five families received at least one home visit. Of them, 50% were ultra-Orthodox Jews and 11% Arab. A high proportion had <12 years education and a third were unemployed. Only 6% of parents lived in separate households. Thirty-eight families had three or more children under the age of five.

Helsinki approval was obtained through the Hospital Ethics Committee (0029-19-ZIV).

### Data collection

Data collection included analysis of documents, questionnaires developed for this study since aside from one existing relevant questionnaire no relevant tools were found in the literature, in-person semi-structured interviews adapted from CFIR's interview guide tool (https://cfirguide.org/) with both hospital and home visitor teams, and through brief telephone interviews with the participating families:

- *Implementation process*: Meeting notes of pre-implementation meetings conducted with hospital management as well as feedback meetings held with hospital management, nursing staff, and home visitors (*n* = 9); a diary documenting the implementation process compiled by a researcher (LS); and Emergency Department attendance for child injury as well as participation in SHABI extracted from hospital medical records and tracking registries (*n* = 5,105).
- *Inner setting*: Meeting notes from pre-implementation meetings held with hospital management; hospital's mission statement; hospital management's views on SHABI and its decision-making process, and nurses' perceptions on SHABI's recruitment and operating evaluated through semi-structured interviews conducted 8 months from baseline (*n* = 13).
- *Intervention characteristics*: Hospital management, nurses, and home visitors' views on SHABI's design and delivery evaluated through semi-structured interviews (*n* = 18).
- *Implementers' characteristics*: Nurses' recruitment skills and home visitors' skills in engaging families and conducting home visits were evaluated through semi-structured interviews; home visitors' post-visit questionnaire on the challenges faced during the visit (*n* = 233); families' views on home visitors' skills were evaluated through telephone interviews conducted after each visit (*n* = 212) by a researcher (LS); home visitors' confidence in conducting the visits were assessed through semi-structured interviews; and home visitors' awareness of dangers in the home was assessed through a questionnaire asking to list the potential dangers in each home area administrated before the first training and 8 months later (*n* = 8), adapted from Kendrick (10).

### Data analysis

Semi-structured interviews along with families' post-visit telephone interviews were recorded and transcribed. All data were analyzed through explanatory content analysis (33) based on CFIR constructs (29). To achieve interrater reliability, two researchers validated the analysis (LS and SS) to ensure the trustworthiness of the results. In case of disagreement, further discussions were held until agreement was reached.

Potential dangers at home were categorized into injury categories and counted for potential dangers reported. Descriptive statistics were used to describe Emergency Department attendance and participation in SHABI. Comparisons of percentages between different groups were analyzed using *X*^2^. Non-normally distributed data were analyzed using Wilcoxon test for dependent subgroups (using SPSS version 27.0).

## Results

Analysis of the data showed a variety of factors affecting SHABI's implementation through the prism of CFIR: the implementation process, the hospital's inner setting, SHABI's characteristics and nurses and home visitors' perceptions and skills. Data is presented in Table 1 according to the themes that emerged and exemplified through relative quotes from hospital management, nurses, home visitors, and families.

**Table 1:** Principal findings regarding the facilitators and barriers in implementing SHABI in the hospital setting.

**CIFR domain and themes**	**Barriers**	**Facilitators**	**Quotes ([+]=facilitator, [-]=barrier)**
**Implementation process**			
Families and home visitors' recruitment process and adherence to the program	773 eligible families arrived at the Emergency Department due to child injury; only 63% were approached by nurses to participate in SHABI Families often failed to agree to participate or be contacted as they felt they had no need for the intervention Less Arab families completed both visits (7 of 15 Arab families completed both visits vs. 91 of 120 Jewish families; *p* = 0.02)	Separation between families' recruitment (done in hospital by nurses) and the home visit components (coordinated by the medical school)	[-]“The mother said there is no safer home than her own and no need for a visit” (Home visitors' post-discharge recruitment phone call to a Jewish mother of two preschoolers from a low SES city)
**Inner setting**			
Compatibility of the hospital's vision with SHABI's mission The hospital's barriers in operating in the community	Despite its mission statement and the hospital director's views on responsibility to the community, in reality, hospital management encountered difficulties in extending its role to the community and operating outside of the hospital setting While recruitment was partially successful in the hospital, concerns about staff insurance outside the hospital precluded hospital nurses conducting home visits as originally intended	SHABI's mission in promoting health in communities located in the hospital catchment area aligned with the hospital's declared mission In the light of hospital barriers, the medical school stepped in and took responsibility for delivery of the home visitation service, and recruited medical and nursing students as home visitors	[+]“I look at the hospital as a community hospital... As a worldview, I would not reduce my responsibility only to what happens within the hospital. I see a broader responsibility within the community as well” (Hospital director) [-]“We work in the hospital, and cannot provide family medicine, community care. It is two different worlds... Hospital is one thing and community is another” (Head of nursing)
The hospital's top-down decision-making process	Nurses perceived that the head nurse daily reports on recruitment was a form of criticism, and that the SHABI coordinator was hardly involved	The top-down decision-making process obligated the nurses to recruit ensuring that it was part of their job	[+]“We received an explanation at the staff meeting with all the managers. We were given an explanation about the program- what was required of us. It is clear to me that this is not democracy, I do not choose what to do at my workplace, it is part of the job” (Nurse #4).
Strategy and available resources	The lack of time in a busy Emergency Department and burdensome nursing tasks affected nurses' ability to recruit	Including recruitment as an additional task in the initial patient triage process facilitated recruitment	[-] “The problem is that SHABI takes time-this is another form that needs to be filled out, and there are many other things that need to be done. There are more people waiting” (Nurse #1)
**Intervention characteristics**			
Recruitment following an injury	Nurses perceived some families were too agitated about their child injury to be approached	Recruitment immediately after a child's injury was perceived to be a definite motivator for parents to consent to SHABI and to actively make changes to their homes	[+]“The parents were very happy that I arrived and wanted to schedule the visit. Both parents were present. They encountered a serious incident [injury] with their daughter, and now are dedicated to prevent similar incidents in the future” (Home visitor #5)
SHABI as a bridge between the hospital and the community		In Israel, the hospital and community interfaces operate independently. Hospital management perceived SHABI as an appropriate bridge between hospital care and preventative community efforts	[+] “I think it is the connection, the connection point, between what we do in the hospital when a child arrives after a home injury, and what happens in the community” (Head of nursing)
Home-visitation intervention design	Home visitors perceived that at times the home tour was felt to be invasive by families	Hospital management, home visitors and families generally perceived that the visit was effective in improving home safety. This drove the home visitors to invest in the intervention The checklist helped to guide the visit and home visitors to discuss safety in each home area	[-] “I felt it (house tour) was an invasion of their privacy. I mean, if the bedroom is messy and the parent does not feel comfortable with it, then it hurts his/her ability to open up to me or listen to the things I want to say. A tour through the home has disadvantages… It can create antagonism” (Home visitor 4#)
**Implementers' characteristics**			
Perception of SHABI's importance	Nurses prioritized their efforts in recruiting families arriving with fall injuries (345 of 508 families with fall injuries were recruited vs. 163 who were not recruited; χ2 = 15.3, *p* < 0.001) in comparison to a foreign body (58 of 119 families with foreign body were recruited vs. 61 who were not recruited; χ2 = 12.2, *p* < 0.001) or due to animal injuries (e.g., dog bite; 23 of 67 families with animal injuries were recruited vs. 44 who were not recruited; χ2 = 25.8, *p* < 0.001)	Nurses perceived SHABI as important which was a significant driver to recruiting families in the Emergency Department	[+]“The [nursing] team members need to build the passion for it [recruitment]... and it also depends on the team member. If they are passionate, it will be more successful” (Nurse #1)
Communication skills	Paucity of Arab speaking home visitors may have influenced communication with Arab families	While nurses and home visitors worried that SHABI's visits might be perceived as judgmental and critical, their sensitivity and explanations that injuries are common allowed constructive engagement	[+]“I explain again and again that it is not a matter of you being a bad parent. There is not a single child that goes through childhood without something happening to him. And it is good to avoid next time” (Nurse # 4) [+]“[The home visitor] was very pleasant, gave a good feeling and did not give a critical and judging feeling, but a sense of sharing and togetherness” (A Jewish mother of three preschoolers from a low SES city)
Home visitors' training	Home visitors had difficulty in encounters with culturally diverse families	Through training and encountering families, home visitors increased their understanding about cultural and religious groups with whom they had little familiarity	[+]“The program completely changed my stereotype. I came from the center of the country to a city like Safed, a low socio-economic city and people with a different background than mine… and it changes something. You suddenly see the person. You do not see he is ultra-Orthodox” (Home visitor #3)
Home visitors' awareness of potential dangers in the home		Increasing awareness influenced home visitors to recognize potential dangers in the home from baseline and 8 months later [6 (IQR 5–7)] vs. [8 (IQR 7–8); *p* < 0.05]	
Home visitors' self-confidence in conducting the visits		The improvement in home-visitors' self-confidence, which was low at the beginning but improved with experience, influenced the visits' effectiveness	[+] “It took a while until I learned how to conduct the conversation [first family phone call] and gain confidence. At first, I would only do it in front of the computer, with the text in front of me and only when there is no noise around me. Now I do it on the go... I initially had the challenge of my insecurity” (Home visitor #1)
Home visitors' skills in conducting the visits	The presence of children distracted from home visitors' ability to conduct the visit	Focusing on building relationships, rather than immediately discussing home safety, enhanced parents' engagement Involving the children in the visit kept them occupied and secured parents' attention	[+]“I kept trying to involve the kids in the visit. I say to the kids: who knows what a door stopper is' [door slamming prevention accessory]? And put it on their noses. 'Who can guess what this product does?' ” (Home visitor #3)

SHABI was designed as a hospital-led program, and its implementation faced several barriers and likewise, facilitators. Analysis indicated that despite the compatibility between SHABI's mission in preventing child injuries and the hospital mission in increasing community health, the hospital found it difficult to operate SHABI outside of its own setting as planned as well as hiring Emergency Department nurses as home visitors. As a result, the medical school took over SHABI's operational aspects and recruited medical/nursing students as home visitors. This collaboration between the hospital and the medical school helped bridge the gap.

SHABI's implementation was facilitated by the top-down decision-making process and nurses perceived SHABI's importance in preventing child injury. Despite the inclusion of recruitment to SHABI in the initial patient triage process, it was still only partially successful. Nurses approached only 63% of eligible families and failed to recruit foreign body or animal injury cases.

Medical and nursing students were recruited as home visitors. Both medical and nursing student cohorts had very few Arabic speakers and none applied for the position. This lack of Arabic speakers may have influenced attrition of Arab families, who were more likely to drop out after the first home visit than Jewish families (7 of 15 Arab families completed both visits vs. 91 of 120 Jewish families; *p* = 0.02). During SHABI's operation and following training sessions, home visitors increased their awareness of dangers at home from baseline and 8 months later [6 (IQR 5–7)] vs. [8 (IQR 7–8); *p* < 0.05]. They also improved their confidence in conducting home visits and enhanced their understanding of cultural and religious groups with whom they had little familiarity. Finally, children's presence in the visits often drew parents' attention, and home visitors involving them in the visit helped reduce distractions.

## Discussion

SHABI is a home-visitation program that aims to prevent unintentional childhood injuries through delivery of a hospital-based service. This study's goal was to evaluate the barriers and facilitators of implementing SHABI using the theoretical and conceptual framework of CFIR (29), exploring different stakeholders' experiences-families and implementers, to better understand the implementation process and outcomes.

Hospitals are an important setting for child injury prevention considering the high arrival and admission rates. Review of hospital-led interventions revealed only two home-visitation studies focused on home safety and injury rate (17–20), however the hospitals' responsibility and involvement remained unclear. This case study contributes to the literature by demonstrating and evaluating the ambiguity regarding the hospital's role and responsibility in implementing SHABI. In the early implementation stages the hospital expressed structural difficulties in operating SHABI outside of its setting as well as in hiring nurses as home visitors. Unlike health systems in other countries, in Israel, hospital and community care settings operate separately using different computerized documentation systems and lacking the mechanisms to mediate between the two (34, 35). To mediate this in SHABI, the collaboration between the hospital and medical school served as a bypass for that structural barrier between hospital and community.

The use of this bypass to overcome the disconnect between the hospital and community was implemented in the ETGAR program (36) aimed at reducing recurrent admissions following discharge from hospital. ETGAR, also developed by the medical school, uses medical students to visit patients and provide guidance following discharge (36). As demonstrated by ETGAR, there is a need for improved coordination and collaboration between hospital and community. Literature suggests that there is specific value for bridging hospital-community silos to the field of child injury prevention. Towner and Dowswell (37) reviewed child injury prevention interventions and found that collaboration between organizations can create an environment in which multiple players, such as municipalities or voluntary agencies, contribute their resources, namely knowledge, experience, or ability, and assist each other when encountering a barrier (37). Despite the benefits of collaborations, as demonstrated in the SHABI program, the bypass created by the hospital and medical school provides only a temporary solution. The structural difficulties of hospitals' involvement in community-hospital prevention programs emphasize the need for designing a sustainable solution that will enable hospitals to become major actors actively contributing to various prevention fields.

Albeit SHABI's recruitment being successful to an extent, one of the organizational catalysts for its implementation was the hospital's top-down decision-making process. Top-down decision-making characterizes hierarchical and clinical implementation settings such as hospitals and Emergency Department (38). Decision-making of this kind can be an influential element in implementing new programs and was found as a motivator for implementers, yet it can also lead to resistance (39, 40). Implementers' beliefs about an intervention serve as an additional significant facilitator for implementation success (41), including staff attitudes regarding hospital-based interventions (42). For example, Garbutt et al. (43) evaluated implementers' beliefs regarding a US national program for papilloma virus vaccines among at-risk girls. They found that implementers who achieved high vaccination rates were those who held a strong belief on the vaccine's importance, who felt self-efficacy and confidence in the vaccine contribution, and were personally committed to the mission. Efforts must therefore be invested in educating implementers about a program's importance in order to create a sense of ownership and achieve sustainable change. Despite some difficulties in accepting the hierarchical decision process, our nurses perceived SHABI as a valuable program, and made efforts to persuade parents to participate.

SHABI's implementers included Emergency Department nurses and home visitors comprised of medical and nursing students rather than only Emergency Department nurses as originally planned. We found that home visitors increased their self confidence in conducting home visits, as well as their awareness toward dangers at home. Along with the significant improvement found in home safety, it seems that professional qualification is not an essential component, and home visitors with adequate training do not harm the program's outcome measures. In the child injury field, several home-visitation studies have used both professional (10, 18) and non-professionals (17, 44, 45) as home visitors. Conflicting findings were found as to home safety increase and/or injury rates decrease, but the literature is unclear as to the necessity for professional qualifications. Further research is needed regarding implementers' required qualification, characteristics, and skills.

Arab families have relatively high levels of injuries in the home (31, 46) and were therefore key targets. Recruitment was lower and there was greater drop out after the first SHABI home visit. This might have been mitigated if the home visitors had among them Arab speaking students. Smithson et al. (27) found that a major barrier to preventing child home injuries is messages that are often not culturally adapted. However, home-visitation interventions where locals were employed as home visitors failed to show significant improvement in home safety and/or injury rate (44, 45). Further research is needed to understand the distinctive skills and characteristics child injury prevention implementers require.

Two opposing forces act simultaneously in the implementation field (21, 22). Fidelity is the need to maintain uniformity according to the original research protocol, compared to adaptability which is the need for protocol adaptation in new settings and contexts to increase implementation success (25, 47, 48). In SHABI, for example, adaptive mechanisms were applied on several occasions. During SHABI's Emergency Department implementation, families' recruitment component was included as part of the patient triage process to ensure that eligible families are included. Additionally, the home visitors developed a strategy for including the children to keep parents' attention during the home visit. Yet the process changes described had no major structural implications to SHABI's core program, did not affect the programs' aspired outcome measures, but maybe increased SHABI's implementation success. Adaptive mechanisms are important as by applying them failure of the implementation process may be prevented (48).

Another aspect in child injury is the vast and diverse existing data on injury prevention. This variability is expressed in several ways, such as dangers in different home areas (kitchen vs. the bedroom), or different injury mechanisms (poisoning vs. burns). This also leads to differences in safety guidelines provided, such as improving the physical environment vs. changing parental behavior or recommending safety devices vs. moving objects out of the child's reach (28). The variance creates diversity in research tools that evaluate effectiveness (8, 49, 50). The lack of uniformity of injury prevention messages, measurement and evaluation tools creates difficulty in developing standards and quality indicators. This difficulty is particularly evident in the attempt to scale-up successful interventions to other settings or larger population groups (24).

Home safety checklists are a common research tool used in child injury prevention, but they generally have not undergone a validation process (17, 44, 45). In SHABI we used a checklist developed by “Beterem” (28), which although based on the literature, has not been formally validated. The HOME inventory (The Home Observation for Measurement of the Environment) (51) appears to be the only validated tool, however only eight out of the 219 items assess home safety, while the rest examine topics such as child physical and emotional development or parent-child attachment. In SHABI we chose the “Beterem” checklist since it has been used widely in Israel. There is no doubt that there is a need to develop validated research tools and standards of quality indicators in the field of child injury.

There are several limitations to this study. The insights gained result from study of a specific clinical setting in the Israeli health system. Further studies are needed in other hospitals in Israel and beyond using various methods and theoretical frameworks in order to extend the conclusions. There was a disparity between the numbers of Arab and Jewish families included in the research population, although the figures reflect the sociodemographic of the hospital's catchment area where the population is 20% Arab. Nonetheless, the lack of Arabic speakers among the home visitors may have reduced SHABI's accessibility to Arab families. It would have been of interest to explore home visitors' attitudes toward local population groups prior to the intervention particularly as the focus on cultural sensitivity was a strength and home visitors claimed that their cultural competence had increased. Lastly, due to the lack of suitable validated research tools, we developed the tools for the current research. This limitation was mitigated by triangulation of the findings from the hospital management's, implementers', and families' perspectives.

## Conclusions

This is the first time that the Implementation Science lens has been used to explore a hospital-led home-visitation intervention aimed at preventing child injury. The conceptual CFIR theoretical framework focused on the entire implementation process, hospital inner setting, SHABI's characteristic, and nurses and home visitors' characteristics. We found that a sustainable solution is needed to bridge the disconnect between the hospital and the community, so that hospitals can become a key player in preventing child injuries. Nurses and home visitors applied adaptive means to increase SHABI's implementation success in the recruitment process at the hospital and during the home visits. Finally, our work further highlights the need to further explore settings for implementing interventions using home visits to prevent child injury.

## Data availability statement

The original contributions presented in the study are included in the article/supplementary material, further inquiries can be directed to the corresponding author.

## Ethics statement

Helsinki approval was obtained through the Ziv Medical Center Ethics Committee (0029-19-ZIV; 22 October 2017). Written informed consent to participate in this study was provided by the participants' legal guardian/next of kin.

## Author contributions

LS was involved in study planning and intervention design, collecting, analyzing data, and drafting the manuscript. MR was the initiator of the study, involved in study planning and intervention design, and reviewing the manuscript. SS was involved in study planning and intervention design, analysis, and reviewing the manuscript. All authors read and approved the final manuscript.

## Funding

This work was supported by Pratt Foundation, Australia. Pratt Foundation–Israel, PO Box 37052, Jerusalem, Israel.

## Conflict of interest

The authors declare that the research was conducted in the absence of any commercial or financial relationships that could be construed as a potential conflict of interest.

## Publisher's note

All claims expressed in this article are solely those of the authors and do not necessarily represent those of their affiliated organizations, or those of the publisher, the editors and the reviewers. Any product that may be evaluated in this article, or claim that may be made by its manufacturer, is not guaranteed or endorsed by the publisher.

## Abbreviations

SHABI: “Keeping our Children Safe”
in Hebrew: “SHomrim Al BetIchut Yeladenu”
CFIR: Consolidated Framework for Implementation Research.
